# Genome-wide dissection of *PP2* genes reveals *CsPP2-3/5/18* as key regulators of phloem protein deposition and bacterial immunity in *Citrus sinensis*

**DOI:** 10.1093/hr/uhaf333

**Published:** 2025-12-04

**Authors:** Wenshan Dai, Tao Hu, Donglian Huang, Yangyang Qin, Nannan Wei, Huanying Xue, Nian Wang, Min Wang

**Affiliations:** National Navel Orange Engineering Research Center, College of Life Sciences, Gannan Normal University, Ganzhou 341000, China; Jiangxi Provincial Key Laboratory of Pest and Disease Control of Featured Horticultural Plants, Gannan Normal University, Ganzhou 341000, China; National Navel Orange Engineering Research Center, College of Life Sciences, Gannan Normal University, Ganzhou 341000, China; National Navel Orange Engineering Research Center, College of Life Sciences, Gannan Normal University, Ganzhou 341000, China; National Navel Orange Engineering Research Center, College of Life Sciences, Gannan Normal University, Ganzhou 341000, China; National Navel Orange Engineering Research Center, College of Life Sciences, Gannan Normal University, Ganzhou 341000, China; National Navel Orange Engineering Research Center, College of Life Sciences, Gannan Normal University, Ganzhou 341000, China; Citrus Research and Education Center, Department of Microbiology and Cell Science, Institute of Food and Agricultural Sciences (IFAS), University of Florida, Lake Alfred, FL, USA; National Navel Orange Engineering Research Center, College of Life Sciences, Gannan Normal University, Ganzhou 341000, China; Jiangxi Provincial Key Laboratory of Pest and Disease Control of Featured Horticultural Plants, Gannan Normal University, Ganzhou 341000, China

## Abstract

Citrus Huanglongbing (HLB), caused by the phloem-restricted bacterium *Candidatus* Liberibacter asiaticus (*C*Las), is a devastating disease threatening global citrus production. *C*Las infection triggers excessive accumulation of phloem proteins (PPs) that obstruct sieve pores, a dual-edged process potentially restricting pathogen spread while impairing phloem transport. Despite its pathophysiological significance, systematic identification and functional characterization of PPs in citrus, particularly their roles in *C*Las defense, remain unclear. Here, we performed a genome-wide analysis of the *PP2* gene family in the HLB-susceptible sweet orange (*Citrus sinensis*) and identified 26 *CsPP2* genes. Phylogenetic and structural analyses uncovered evolutionary divergence and regulatory complexity among CsPP2 family members. Using promoter-driven *GUS* gene expression assays in transgenic hairy roots, we identified three phloem-specific paralogs, *CsPP2-3*, *CsPP2-5*, and *CsPP2-18*, and delineated core regulatory regions conferring tissue specificity. Overexpression of each gene significantly enhanced phloem protein deposition. Notably, although virus-induced silencing of individual *CsPP2s* did not affect resistance to *Xanthomonas citri* subsp. *citri*, overexpression of any of the three genes substantially enhanced resistance against this apoplastic pathogen, demonstrating functional redundancy. However, the three paralogs exhibited marked functional divergence in response to *C*Las: *CsPP2-3* and *CsPP2-18* conferred enhanced resistance, whereas *CsPP2-5* increased susceptibility. Distinct defense-related gene expression profiles further supported their specialized immune roles. Our study provides the first systematic identification of *PP2* genes in citrus and reveals the functional differentiation of *CsPP2-3/5/18* as key regulators of phloem-mediated defense. These findings provide crucial insights into phloem defense regulatory networks and identify novel genetic targets for HLB resistance breeding.

## Introduction

Citrus, a globally dominant fruit crop ranking third in agricultural trade value, underpins rural economies across production regions [1, 2]. Particularly in southern China, a primary center of citrus origin and diversification, this industry drives economic growth and rural revitalization [3, 4]. However, citrus Huanglongbing (HLB), caused by phloem-restricted *Candidatus* Liberibacter species, now poses an existential threat to global citrus production. Since its emergence, HLB has devastated citrus industries in Florida, Brazil, and major Asian production zones [5]. The predominant pathogen, *Candidatus* Liberibacter asiaticus (*C*Las), colonizes phloem sieve tubes and remains unculturable *in vitro* [6]. *C*Las infection triggers progressive physiological collapse: early symptoms include asymmetric leaf chlorosis and shoot stunting, advancing to characteristic ‘red-nosed’ fruit deformities, branch dieback, and systemic tree decline [7]. Critically, infected trees exhibit increased susceptibility to environmental stresses, such as temperature and humidity changes, accelerating leaf abscission, premature fruit drop, and secondary infections. These pathological manifestations collectively reduce yield by 40% to 100%, shorten orchard lifespans, and compromise fruit quality, incurring significant economic losses to the citrus industry.

Phloem proteins (PPs) are highly complex components of phloem tissue, and their functions remain poorly understood. Previous studies have identified two main types of PPs in phloem sap: phloem protein 1 (PP1) and phloem protein 2 (PP2) [8]. PP1 is a primary structural protein of monomeric fibers that form basal sieve plate fibers in the phloem. In contrast, PP2 acts as a dimeric poly-GlcNAc-binding lectin and has been implicated in various biological processes, particularly plant defense and signal transduction. Recombinant CsPP2-A1 protein from cucumber exhibits hemagglutinating activity and potently inhibits the growth of pathogenic fungi *Botrytis cinerea* and *Phytophthora infestans* [9]. Similarly, AtPP2-A1 performs dual functions, exhibiting both molecular chaperone activity and antifungal activity against pathogens like *Fusarium solani* [10]. Beyond direct antimicrobial effects, PP2 also modulates defense signaling. For instance, AtPP2-A5 was found to confer tolerance to the two-spotted spider mite (*Tetranychus urticae*) by orchestrating transcriptional reprogramming that alters hormone accumulation and signaling pathways. Overexpression or knockout of *AtPP2-A5* disrupts the balance of these key defense hormones [11]. In terms of defense responses, PP2 proteins are also crucial players in abiotic stress responses, often exhibiting complex regulatory roles. The SCF E3 ligase AtPP2-B11 acts as a negative regulator of drought tolerance by degrading AtLEA14 and suppressing stress-responsive genes in *Arabidopsis* [12]*.* Conversely, it functions as a positive regulator of salt tolerance by enhancing annexin-mediated reactive oxygen species (ROS) scavenging and Na^+^ homeostasis [13]. Furthermore, AtPP2-B11 specifically targets SnRK2.3 for 26S proteasomal degradation, revealing a novel negative feedback mechanism to attenuate abscisic acid (ABA) signaling and stress responses [14]. Supporting the importance of PP2 in ABA signaling, loss-of-function of *AFA1* (*AtPP2-B1*) enhances ABA hypersensitivity during seed germination and improves drought tolerance. This occurs through hyperactivation of ABA-responsive genes (e.g. *ABI5*) due to impaired ubiquitin-mediated degradation of positive regulators within the SCF-AFA1 complex [15]. Additionally, PP2 proteins contribute to systemic signaling through their interaction with RNA molecules and participation in the long-distance transport of macromolecules, suggesting a potential role in coordinating responses during plant-pathogen interactions [16–18]. Collectively, the multifaceted roles of PP2s in defense, stress adaptation, hormone signaling, and systemic communication underscore their fundamental importance in plant physiology and underscore the critical need for further in-depth investigation into this versatile protein family.

Following *C*Las infection, characteristic pathological alterations manifest within the phloem, including pronounced starch accumulation, cell wall thickening, sieve tube occlusion, and eventual sieve element collapse [19]. Crucially, PP2 proteins have been directly implicated in these structural disruptions. Immunogold labeling identified filamentous aggregates plugging sieve tubes in HLB-infected sweet orange and grapefruit as PP2 proteins [20]. Furthermore, temporal observations indicate that PP2 deposition constitutes an early response event, preceding starch accumulation, sieve pore blockage, and cell collapse during *C*Las infection [21]. Extensive phloem protein deposition and sieve tube blockage have been observed in HLB-infected sweet orange leaf veins, and gene chip analysis further revealed significant upregulation of *PP2* genes in the phloem of *C*Las-infected sweet orange leaves [22]. Notably, *CsPP2-B15* was identified as one of the most strongly induced markers in HLB-responsive sweet orange, showing a 330.8-fold induction in ‘Valencia’ plants grafted with *C*Las-infected buds [23]. Despite its established role as a transcriptional marker, the functional significance of *CsPP2-B15* and related *PP2* genes beyond their expression patterns remains poorly characterized. Additionally, most studies on the HLB-induced upregulation of *PP2* genes have focused on HLB-susceptible varieties, suggesting that the *PP2* expression and phloem occlusion may contribute to the disease resistance response in these varieties [24]. In conclusion, these evidence strongly suggest that *C*Las-induced *PP2* overexpression and subsequent phloem protein aggregation may represent a host defense mechanism aimed at restricting pathogen spread through phloem occlusion. However, while PP2 deposition is a prominent feature of the HLB response in susceptible citrus, the specific functions of these HLB-associated *PP2* genes and their underlying molecular mechanisms in citrus pathogenesis and defense remain largely unexplored.

In this study, we conducted a comprehensive identification of the *PP2* gene family in *Citrus sinensis*, a representative HLB-susceptible citrus species and characterized the expression dynamics of *CsPP2* genes under *C*Las infection. We systematically investigated the tissue-specific expression patterns of all *CsPP2* promoters, identifying three phloem-localized members (*CsPP2*-3/5/18) and defining their core promoter regions conferring phloem specificity. Functional validation via the *Agrobacterium rhizogenes*-mediated hairy root transformation confirmed these three *CsPP2s* positively regulate phloem protein deposition. Strikingly, virus-induced gene silencing (VIGS) and transient overexpression assays revealed that *CsPP2-3/5/18* exhibit functional redundancy in enhancing bacterial disease resistance, likely through distinct molecular mechanisms. Collectively, our work provides mechanistic insights into HLB-responsive *CsPP2* genes, establishing their roles in phloem occlusion and host defense, and offering valuable genetic targets for HLB resilience breeding.

## Results

### Genome-wide identification and phylogenetic analysis of *PP2* gene family in *C. sinensis*

Comprehensive identification of the *PP2* gene family in *C. sinensis* was achieved through Hidden Markov Model (HMM) profiling using the PP2 domain (PF14299) and subsequent BLASTp screening against the sweet orange proteome. Initial retrieval yielded 37 candidate genes, which were rigorously filtered to remove redundant transcripts and incomplete domain sequences, resulting in 26 high-confidence *CsPP2* genes systematically designated as *CsPP2-1* to *CsPP2-26* according to chromosomal location (Table S1). These genes encode proteins exhibiting remarkable molecular diversity: coding sequences spanned 480 bp (*CsPP2-10*) to 1911 bp (*CsPP2-26*), translating to polypeptides of 159 to 636 amino acids with corresponding molecular weights of 18.12 to 73.02 kDa. Isoelectric point (pI) analysis revealed a bimodal distribution, with 11 acidic (pI 4.91–6.95) and 13 basic (pI 7.07–8.89) isoforms, suggesting functional divergence within the family.

**Figure 1:**
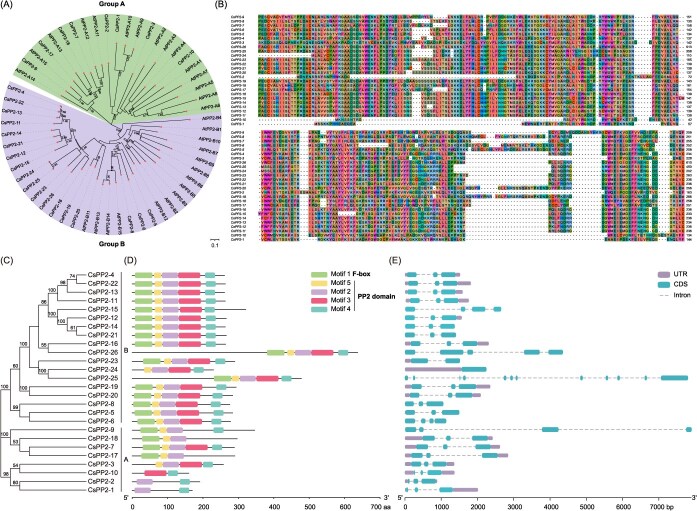
Identification and characterization of *CsPP2* genes. (A) Phylogenetic analysis of *PP2* genes from *C*. *sinensis* (Cs) and *A*. *thaliana* (At). (B) Multiple sequence alignment of the conserved domains in CsPP2 proteins. (C) Phylogenetic relationships of CsPP2 proteins. (D) Distribution of five conserved motifs identified by MEME program. Motif sequences are detailed in Table S2. (E) Exon/intron structures of *CsPP2* genes. CDS, coding sequence; UTR, untranslated regions.

Phylogenetic reconstruction with *Arabidopsis thaliana* orthologues conserved the dichotomous Group A/B classification but uncovered significant lineage-specific divergence. *Arabidopsis* maintained balanced clades (15 AtPP2s per group), while citrus exhibited preferential expansion of Group B (18 CsPP2s) over Group A (Fig. 1A). Multiple sequence alignment confirmed universal conservation of the core PP2 domain across all 26 proteins (Fig. 1B).

### Conserved domain and structural organization of *CsPP2* genes

Gene structure analysis is essential for understanding the relationship between gene evolution and functional differentiation [25]. Five conserved motifs were identified, ranging in length from 21 to 63 aa (Table S2). Motif 1 corresponds to the F-box domain, known to mediate substrate recognition in E3 ubiquitin ligase complexes [26]. Twenty-one CsPP2 proteins in *C*. *sinensis* contain this N-terminal F-box domain, suggesting roles in targeted protein degradation and protein–protein interactions [27]. Motifs 2 to 5 collectively constitute the conserved PP2 domain at the C-terminus, with motif 4 being essential. The motif composition across CsPP2 proteins is illustrated in Fig. S1. Notably, motif architecture is highly conserved among phylogenetically related CsPP2 members. For example, CsPP2-1, -2, -3, and -10 lack the F-box domain entirely (Fig. 1C and D).

Analysis of exon/intron organization across the 26 *CsPP2* genes revealed structural diversity. While the majority (19/26) possess three exons, exceptions include *CsPP2-24* (1 exon), *CsPP2-23* (2 exons), *CsPP2-4/9/15* (4 exons), *CsPP2-26* (5 exons), and *CsPP2-25* (12 exons). In addition, intron/exon positions and structures are generally conserved within phylogenetic clusters. For instance, *CsPP2-14* and *CsPP2-21* from Group A share nearly identical three-exon structures (Fig. 1E).

### Chromosomal location and synteny analysis of *CsPP2* genes

**Figure 2:**
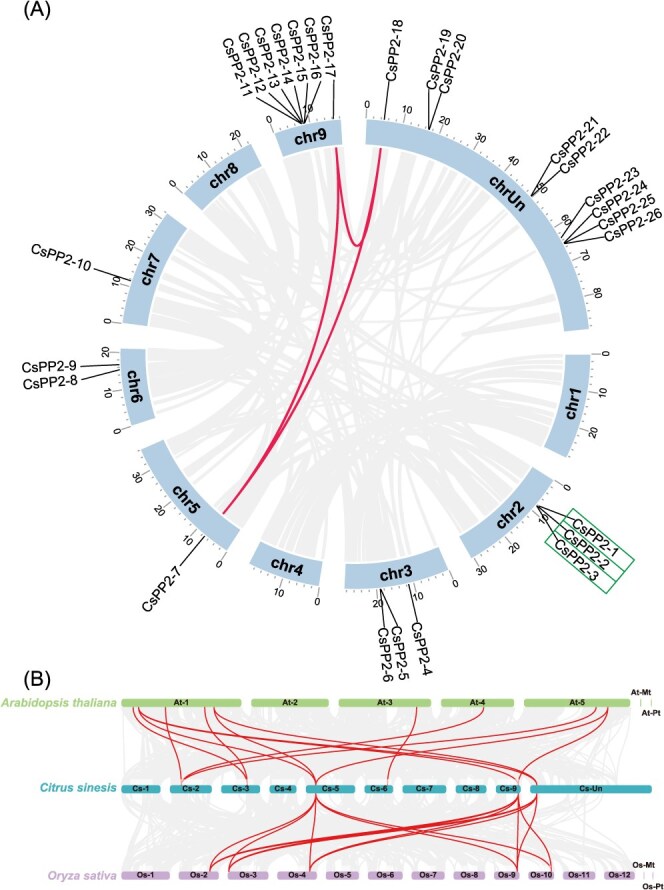
Genomic distribution and synteny analysis of *PP2* genes. (A) Chromosomal distribution and collinearity of *CsPP2* genes. Chromosomes are shown as vertical ideograms (lilac) with scale bars. Gene positions are marked outside chromosomes. Dark curves, segmental duplication pairs; rectangular boxes, tandem duplication clusters. (B) Interspecific synteny of *PP2* genes among *C*. *sinensis, A*. *thaliana,* and *O*. *sativa*. Chromosomes are depicted as horizontal columns, with the chromosome numbers at the center. Light curves, background syntenic blocks; Dark curves, collinear *CsPP2* orthologue pairs

Chromosomal localization of the 26 *CsPP2* genes revealed an uneven distribution across the nine chromosomes of *C*. *sinensis*. Chromosome 9 harbored the highest density (7 genes), followed by chromosomes 2 and 3 (3 genes each). Chromosomes 5, 6, and 7 contained 1 to 2 genes each, while no *CsPP2* genes were detected on chromosomes 1, 4, or 8. Nine genes remained unassigned to specific chromosomes (Fig. 2A). Gene duplication events drive gene family expansion and functional diversification [28]. MCScanX analysis identified three segmental duplication pairs (*CsPP2-7*/*CsPP2-17*, *CsPP2-7*/*CsPP2-18*, *CsPP2-17*/*CsPP2-18*) and two tandem duplication pairs (*CsPP2-1*/*CsPP2-2*, *CsPP2-2*/*CsPP2-3*) (Fig. 2A), suggesting segmental duplication as the primary driver of CsPP2 family expansion in *C*. *sinensis*.

Interspecific synteny analysis with *A. thaliana* (dicot) and *Oryza sativa* subsp. *japonica* (monocot) revealed 13 collinear *CsPP2*-*AtPP2* pairs and 14 *CsPP2*-*OsPP2* pairs (Fig. 2B), indicating deep evolutionary conservation of *PP2* genes. Notably, *CsPP2-7* showed collinearity with eight orthologues across both species. Multiple duplication events among these syntenic blocks suggest functional conservation during *PP2* gene family evolution.

### *cis*-Acting element analysis of *CsPP2* promoters

**Figure 3:**
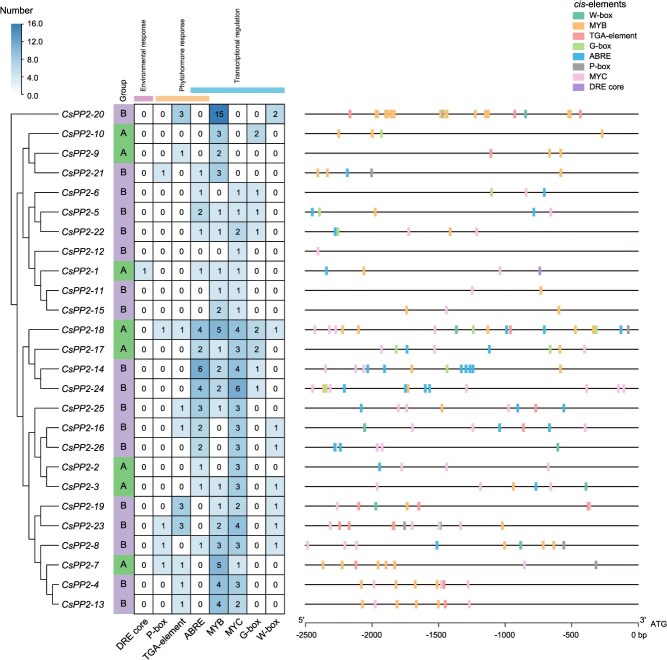
*cis*-acting element profiling in *CsPP2* promoters. *Cis*-acting elements predicted within 2.5-kb promoter regions upstream of transcription start sites. Heatmap indicates normalized abundance of element types across *CsPP2* genes (color scale, low to high). Color-coded blocks represent distinct element categories. Detailed information of these *cis*-acting elements is shown in Table S3.

*cis*-acting elements are specific DNA sequences located in gene promoter regions that are essential for gene expression and play critical roles in regulating plant growth, development, and environmental responses [29, 30]. Systematic scanning of 2.5-kb promoter regions upstream of the transcription start site (TSS) identified 189 functional *cis*-regulatory elements across *CsPP2* genes, excluding core promoter elements (TATA/CAAT boxes) (Fig. 3; Table S3). Element counts per promoter ranged from 1 (*CsPP2-12*) to 20 (*CsPP2-20*). All *cis*-acting elements were classified into three functional categories: (i) 53 phytohormone-responsive elements, dominated by abscisic acid (ABA)-responsive elements (32, 16.93%), auxin-responsive elements (16, 8.46%), and gibberellin (GA)-responsive elements (5, 2.65%); (ii) a single dehydration-responsive element (DRE) exclusively in *CsPP2-1*; and (iii) 135 transcriptional regulatory elements, predominantly MYB transcription factor (TF)-binding elements (60, 31.75%) and MYC TF-binding elements (55, 29.10%), followed by bHLH TF-binding elements (11. 5.82%) and WRKY TF-binding elements (9, 4.76%). Notably, MYB/MYC TF-binding elements occurred in nearly all promoters, while ABRE elements (functional binding sites for ABF transcription factors) showed significant enrichment. Certain *CsPP2s*, such as *CsPP2-*14, -18, -20, and -24, contained diverse hormone-responsive and transcriptional regulatory elements in their promoters, implying their potential for rapid and robust responses to specific hormones or upstream regulatory factors.

### Transcriptional regulatory network of *CsPP2* genes

**Figure 4:**
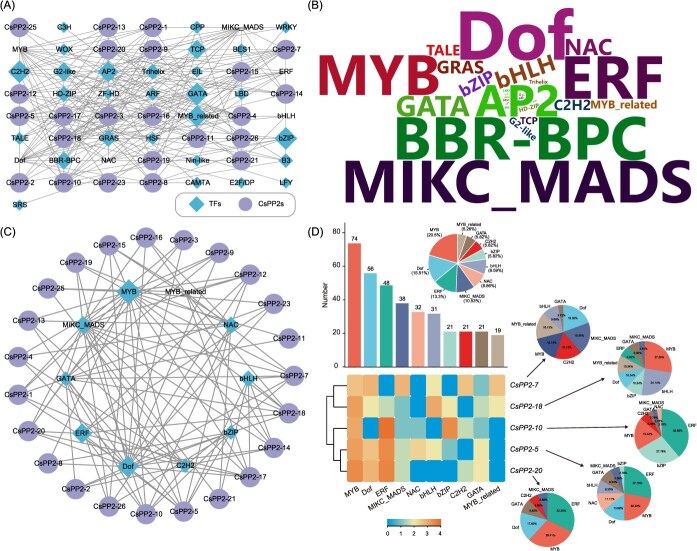
Prediction of transcription factors (TFs) targeting *CsPP2* genes. (A) Correlation network between predicted TFs and *CsPP2* genes. Diamonds, transcription factors (size = target gene number); circles, *CsPP2* genes; lines, connectors (thickness = correlation strength). (B) TF abundance wordcloud. Font size scales with the number of TF representatives. (C) Top 10 TF families predicted to regulate *CsPP2* genes. (D) Top 5 *CsPP2* genes by predicted regulator count. Detailed information of predicted TFs is described in Table S4.

Based on *cis*-acting element profiling, we constructed the upstream regulatory network for *CsPP2* genes. 198 transcription factors (TFs) from 34 families were predicted to regulate *CsPP2s* (Fig. 4; Table S4). The MYB family (74 members) represented the most abundant, followed by Dof (56), ERF (48), MIKC_MADS (38), NAC (32), bHLH (31), and bZIP (21). In contrast, the least abundant TF families, such as ZF-HD (1), SRS (1), and EIL (2), contained only a few members. According to the prediction results, *CsPP2-10* possessed the highest number of regulators (52), followed by *CsPP2-5* (46) and *CsPP2-7* (43) (Fig. 4A and B; Table S4). Furthermore, we identified 10 TF families predicted to regulate all *CsPP2s*, including MYB, Dof, ERF, MIKC_MADS, NAC, bHLH, bZIP, C2H2, GATA, and MYB_related (Fig. 4C). These TF families were found to be significantly enriched and likely play a crucial role in the transcriptional regulation of *CsPP2* genes in *C*. *sinensis*. Further analysis revealed that the top five *CsPP2* genes regulated by the most TFs were *CsPP2-5* (36), *CsPP2-10* (36), *CsPP2-20* (34), *CsPP2-7* (31), and *CsPP2-18* (29) (Fig. 4D). Notably, these predictions were consistent with the results of the promoter *cis*-acting element analysis. For example, *CsPP2-20*, which contained the highest number of *cis*-acting elements in its promoter, was predicted to be regulated by TFs such as ERF, MYB, Dof, GATA, C2H2, and MIKC_MADS, consistent with the types and numbers of *cis*-acting elements present in its promoter. Overall, the predicted TF regulatory network of *CsPP2s* suggests their potential roles in plant growth, hormone signaling, environmental stress responses, and network interactions.

### Expression profiles of *CsPP2s* under *C*Las infection

**Figure 5:**
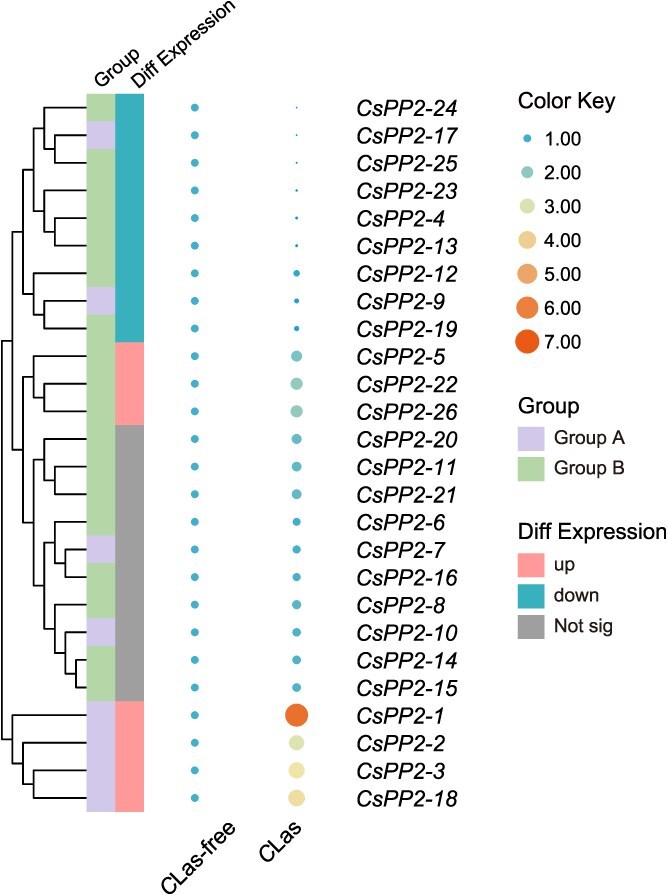
Differential expression of *CsPP2* genes in response to *C*Las infection. qRT-PCR data were normalized by log2 transformation and visualized as a heatmap using TBtools. The clustering algorithm adopted is complete linkage clustering (furthest neighbor clustering). A vertical color scale (right) indicates expression levels.

Previous studies established that *C*Las, a phloem-restricted parasitic bacterium, induces massive deposition of phloem proteins within citrus sieve tubes [31]. Leaves of healthy (*C*Las-free) and symptomatic HLB (*C*Las) of *C. sinensis* with uniform developmental stage were collected from citrus groves (Fig. S2A). Transmission electron microscopy (TEM) was subsequently performed following quantitative reverse transcription-PCR (qRT-PCR) validation of *C*Las infection status, based on *16S* gene cycle threshold (Ct) values (Fig. S2B). TEM images of symptomatic leaf veins confirmed *C*Las colonization within sieve tubes (blue arrows, Fig. S2C), concomitant with extensive phloem protein deposition manifesting as filamentous aggregates (red arrow, Fig. S2C). Subsequent qRT-PCR profiling of 26 *CsPP2* genes in *C*Las-infected versus healthy midribs revealed divergent expression patterns. Ten *CsPP2* genes (*CsPP2-6*/*7*/*8*/*10*/*11*/*14*/*15*/*16*/*20*/*21*) exhibited no significant expression changes following *C*Las infection, while 16 exhibited significant differential expression. Notably, seven genes (*CsPP2-1/2/3/5/18/22/26*) were upregulated and nine (*CsPP2-4/9/12/13/17/19/23/24/25*) were downregulated in symptomatic leaves (Fig. 5; Table S5).

### Phloem-specific expression profiling of the *CsPP2* promoters

**Figure 6:**
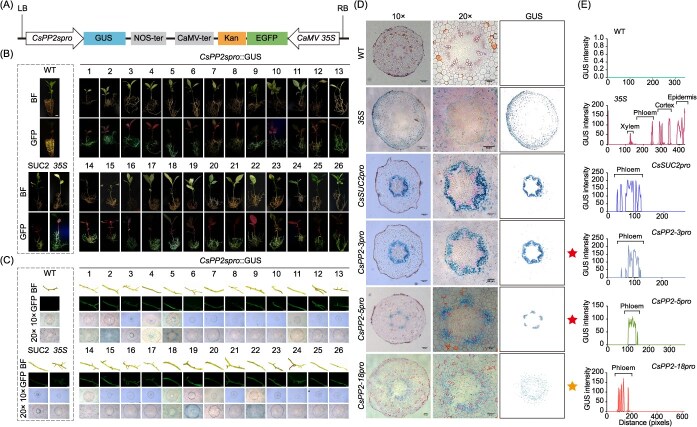
*A*. *rhizogenes*-mediated transformation and phloem-specific promoter activity of *CsPP2*. (A) Schematic of *CsPP2spro/CsSUC2pro/35S*::GUS-GFP reporter vector. (B) Transformation validation. Bright field (BF) and GFP fluorescence images of wild-type (WT) vs. transgenic hairy roots. Phloem-specific positive control, *CsSUC2pro*; Positive control, *35S*. Scale bar = 1 cm. (C) Fluorescence microscopy and GUS staining of root cross-sections. (D) High-resolution GUS staining (10×, 100 μm scale; 20×, 50 μm scale). Pentagrams, phloem-specific signals. Right panels, ImageJ-isolated GUS signals from 10× views. (E) Quantified GUS activity in vascular tissues using ImageJ.

Although phloem protein deposition following HLB infection has been widely reported, the responsible *CsPP2* members remained unknown. To elucidate specific CsPP2 members mediating *C*Las-induced phloem protein deposition, we systematically investigated the tissue specificity of all 26 *CsPP2* promoters. The 2.5-kb promoter regions upstream of the TSS for all 26 *CsPP2* genes were cloned into a dual-reporter vector containing GUS and GFP tags, generating *CsPP2spro*::GUS constructs (Fig. 6A). These constructs were stably transformed into *C. sinensis* via *Agrobacterium rhizogenes*-mediated hairy root transformation [32], with three distinct controls: (1) *CsSUC2pro*::GUS for phloem-specific positive control [33]; (2) *35S*::GUS for positive control; and (3) wild-type (WT, non-transformed) roots as negative control. Successful transformation was confirmed by GFP fluorescence in all transgenic roots (Fig. 6B). Subsequently, the positive transgenic roots emitting green fluorescence were selected for GUS staining and sectioned for microscopic observation. Histochemical GUS staining revealed three distinct expression patterns: (i) no detectable activity (*CsPP2-1pro*, *CsPP2-8pro*, *CsPP2-9pro*, *CsPP2-14pro*, and *CsPP2-24pro*); (ii) constitutive pan-tissue expression (*CsPP2-2pro*, *CsPP2-4pro*, *CsPP2-19pro*, *CsPP2-23pro*, and *CsPP2-25pro*) resembling *35S*::GUS controls; and (iii) phloem-specific expression vascular bundles (*CsSUC2pro*, *CsPP2-3pro*, *CsPP2-5pro*, and *CsPP2-18pro*) (Fig. 6C–E). In particular, *CsPP2-3pro* and *CsPP2-5pro* exhibited robust phloem-specific GUS activity (red pentagrams, Fig. 6D), whereas *CsPP2-18pro* displayed weaker yet phloem-confined signals (orange pentagram, Fig. 6D). Interestingly, despite *CsPP2-1* exhibiting the most pronounced up-regulation (86.0-fold) following *C*Las infection (Fig. 5), its promoter did not yield discernible GUS activity. To circumvent the notoriously poor rooting capacity of *C*Las-infected *C. sinensis*, we further assayed the *CsPP2-1pro* in *C*Las-infected lime (*Citrus aurantifolia*) branches to confirm its phloem specificity (Fig. S3A and B). Histochemical analysis revealed that *C*Las-infected hairy roots transformed with *CsPP2-1pro*::GUS-EGFP exhibited intense and ubiquitous GUS signals across all tissue types (Fig. S3C–E), demonstrating no phloem-specific expression characteristics of *CsPP2-1pro*, even under *C*Las infection. Taken together, these results establish that *CsPP2-3*, *CsPP2-5*, and *CsPP2-18* possess functional phloem-specific promoters, implicating them as primary mediators of phloem protein accumulation during HLB pathogenesis.

### Identification of phloem-specific regulatory regions in *CsPP2-3*/*5*/*18* promoters

**Figure 7:**
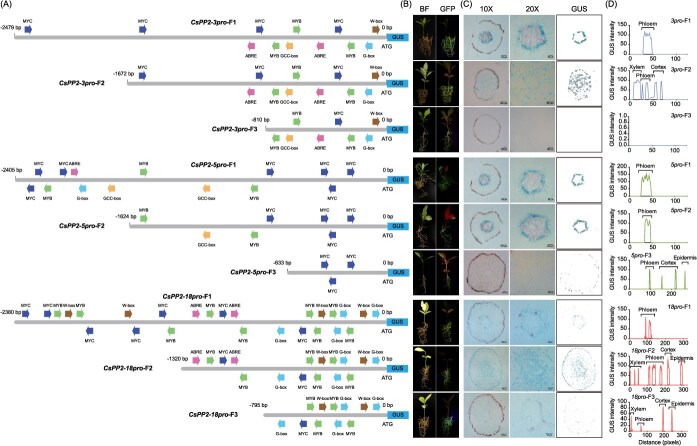
Functional mapping of phloem-specific regulatory regions in *CsPP2–3*/*5*/*18* promoters. (A) Schematic of full-length (FL) and truncated *CsPP2–3/5/18* promoters. Colored arrows represent distinct *cis*-acting elements. (B) GFP fluorescence in transgenic hairy roots. (C) GUS-stained cross-sections of transgenic hairy roots (10×, 100 μm scale; 20×, 50 μm scale). The rightmost column displays GUS-stained regions isolated from 10× magnification views using ImageJ. (D) The GUS intensities of cross-sections in transgenic hairy roots.

To delineate minimal regulatory regions conferring phloem specificity, we generated truncated promoter fragments for *CsPP2-3*, *CsPP2-5*, and *CsPP2-18* based on *cis*-acting element distribution (Fig. 7A). Three truncations per promoter (designated F1–F3) were stably transformed into *C. sinensis* hairy roots (Fig. 7B). GUS staining and quantitative intensity analysis showed that for *CsPP2-3pro,* F1 (full-length) exhibited phloem-specific expression, whereas F2 showed ubiquitous signals throughout root tissues and F3 abolished activity, localizing the essential regulatory region to −2479 to −1672 bp. For *CsPP2-5pro*, both F1 (full-length) and F2 (N-terminal truncation) retained phloem specificity, but F3 eliminated tissue-specific expression, confining the functional domain to −1624 to −633 bp. Similarly for *CsPP2-18pro*, F1 displayed phloem-specific signals while F2 lost tissue specificity, and F3 further reduced activity, indicating critical regulatory elements within −2380 to −1320 bp (Fig. 7C and D).

### Expression dynamics and subcellular localization of *CsPP2-3*/*5*/*18*

**Figure 8:**
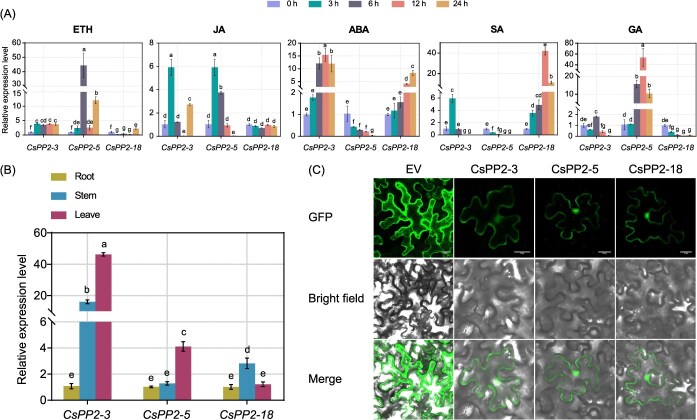
Expression pattern and subcellular localization of *CsPP2–3*/*5*/*18*. (A) Relative expression levels of *CsPP2-3/5/18* in *C*. *sinensis* leaves treated with ETH, JA, ABA, SA, or GA at 0, 3, 6, 12, and 24 h. (B) Tissue-specific expression profiles in roots, stems, and leaves of *C*. *sinensis*. Different lowercase letters above the bars indicate significant differences (Tukey’s test, *P* < 0.05). (C) Subcellular localization via *Agrobacterium*-mediated transient expression in tobacco epidermal cells. Fluorescence was captured by a confocal laser scanning microscope; scale bars = 20 μm.

Promoter analysis revealed enrichment of phytohormone-responsive *cis*-acting elements in *CsPP2-3/5/18*, implying potential roles in hormone-mediated processes. To functionally validate this, we quantified their transcriptional responses to five hormones, including ethrel (ETH), jasmonic acid (JA), abscisic acid (ABA), salicylic acid (SA), and gibberellin (GA), via qRT-PCR. *CsPP2-3* was upregulated by all five hormones, peaking at 15.4-fold induction under JA, suggesting broad integration of phytohormone signals. *CsPP2-5* exhibited antagonistic regulation, induced by ETH/JA/GA but suppressed by ABA/SA, indicating potential balancing roles in SA/JA defense pathways. *CsPP2-18* showed downregulation by ETH/GA, upregulation by ABA/SA, and no significant change under JA (Fig. 8A). Furthermore, tissue-specific profiling identified predominant *CsPP2-3* expression in stems/leaves, leaf-specific *CsPP2-5* accumulation, and stem-preferential *CsPP2-18* expression (Fig. 8B). Finally, transient expression in tobacco confirmed nuclear and plasma membrane localization for all three proteins, with distinct fluorescent signals observed in both compartments (Fig. 8C).

### Effects of *CsPP2-3*/*5*/*18* overexpression on phloem morphology and phloem protein deposition

**Figure 9:**
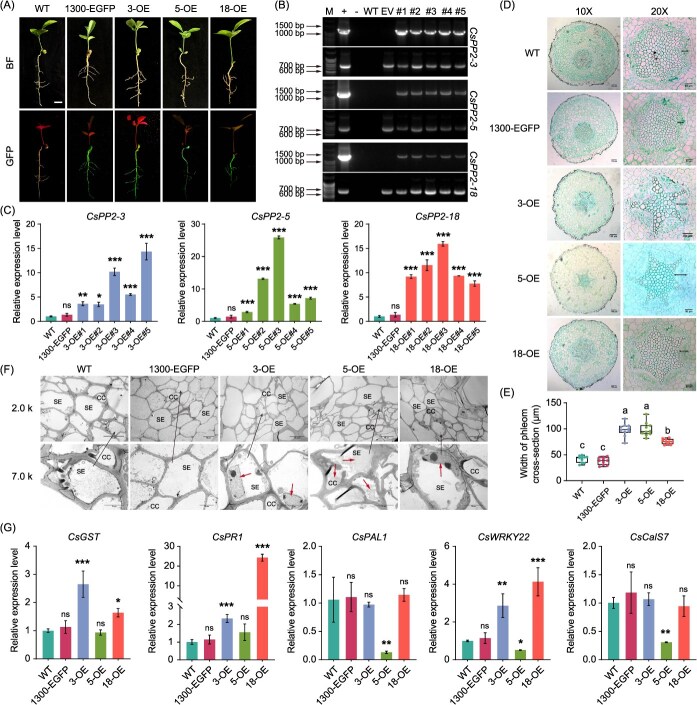
Overexpression of *CsPP2–3/5/18* alters phloem morphology and protein deposition in *C*. *sinensis* hairy roots. (A) Phenotypes of WT, empty vector control (EV, 1300-EGFP), and *CsPP2–3/5/18*-overexpressing hairy roots under BF and GFP fluorescence. Scale bar = 1 cm. (B) Genomic PCR validation of transgenic lines. Upper panel, CaMV *35S*/gene-specific primers. Lower panel, *GFP*-specific primers. +, plasmid (positive control); −, ddH_2_O; WT, wild-type; EV, empty vector (1300-EGFP); #1–5, independent transgenic lines. (C) Relative transcript levels of *CsPP2–3/5/18* in transgenic lines versus WT roots. Data represent mean ± SD (*n* = 3 biological replicates). Asterisks indicate significant differences compared to WT (Tukey’s test; ^*^, *P* < 0.05; ^**^, *P* < 0.01; ^***^, *P* < 0.001). (D) Representative root cross-sections. 10×: 100 µm scale; 20×: 50 µm scale. The dark horizontal lines indicate the thickness of the phloem. (E) Quantification of phloem thickness. Values represent mean ± SE (*n* ≥ 10 roots/group). Different lowercase letters above bars denote significant differences (Tukey’s test; *P* < 0.05). (F) TEM images of sieve elements. Short arrows indicate phloem protein deposits. PC, parenchyma cells; SE, sieve element; CC, companion cells. Scale bars, 50 μm (2.0 k) or 20 μm (7.0 k). (G) Relative expression of defense-related genes. Data represent mean ± SD (*n* = 3 biological replicates). Asterisks indicate significant differences compared to WT (Tukey’s test; ^*^, *P* < 0.05; ^**^, *P* < 0.01; ^***^, *P* < 0.001). ns, not significant.

To further characterize the functional roles of *CsPP2-3*/*5*/*18*, we employed *A*. *rhizogenes*-mediated transformation to overexpress these genes in sweet orange seeds (Fig. 9A). Genomic PCR of transgenic roots using two specific primer pairs confirmed successful integration of the overexpression constructs (Fig. 9B). qRT-PCR analysis revealed substantial upregulation of *CsPP2-3*/*5*/*18* transcripts in transgenic compared to WT controls, with expression levels ranging from 2.88-fold (5-OE#1) to 25.88-fold (5-OE#3) (Fig. 9C). Overexpression of *CsPP2-3/5/18* significantly increased phloem thickness relative to WT roots, whereas roots transformed with the empty vector (1300-EGFP) showed no such effect (Fig. 9D and E). TEM observation further demonstrated enhanced deposition of filamentous or flocculent phloem protein in sieve tubes of *CsPP2-3*/*5*/*18* overexpressing lines (Fig. 9F), indicating these genes promote phloem protein accumulation in phloem cells of sweet oranges. Notably, defense-related gene expression diverged among overexpressors. *CsPP2-3* and *CsPP2-18* overexpressing lines exhibited significant upregulation of *CsGST*, *CsPR1*, and *CsWRKY22*, while *CsPP2-5*-overexpressing roots showed downregulation of *CsPAL1*, *CsWRKY22*, and *CsCalS7* (Fig. 9G). This suggests that despite their shared role in phloem protein deposition, *CsPP2-3*, *CsPP2-5*, and *CsPP2-18* differentially modulate citrus immune responses, potentially reflecting specialized functions in HLB resistance.

### Functional redundancy among *CsPP2-3/5/18* in disease resistance

**Figure 10:**
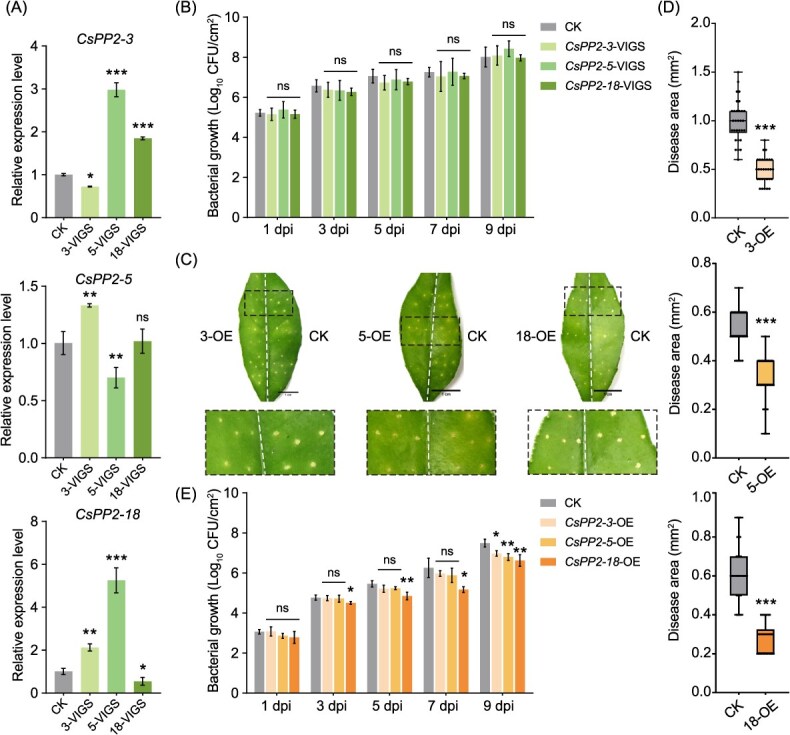
Functional redundancy among *CsPP2–3/5/18* in modulating bacterial resistance in *C*. *sinensis*. (A) Transcript levels of *CsPP2–3/5/18* at 3 dpi in leaves subjected to VIGS targeting individual paralogs. Expression in the empty vector control (CK) is set to 1. Data represent mean ± SD (*n* = 3 biological replicates). Asterisks indicate significant differences compared to CK (Tukey’s test; ^*^, *P* < 0.05; ^**^, *P* < 0.01; ^***^, *P* < 0.001). (B) *Xcc* growth kinetics in VIGS-silenced lines. Bacterial titers (log_10_CFU/cm^2^) were quantified at 1, 3, 5, 7, and 9 dpi. Data represent mean ± SD (*n* = 3 biological replicates). ns, not significant. (C) Disease phenotypes at 9 dpi following *Xcc* needle-prick inoculation of leaves transiently overexpressing individual *CsPP2* genes. Insets show magnified views of necrotic lesions (dashed boxes). CK, empty vector control; 3-OE, 5-OE, and 18-OE, *CsPP2–3*, *CsPP2–5*, and *CsPP2*–*18*-overexpressing lines, respectively. Scale bars = 1 cm. (D) Quantification of disease area in *Xcc*-inoculated leaves at 9 dpi. Lesion areas were measured using ImageJ software, with at least three leaves and ten lesion spots per leaf analyzed. (E) *Xcc* titers (log_10_CFU/cm^2^) in overexpression lines at 1, 3, 5, 7, and 9 dpi. Data represent mean ± SD (*n* = 3 biological replicates). Asterisks indicate significant differences compared to CK (Tukey’s test; ^*^, *P* <0.05; ^**^, *P* < 0.01). ns, not significant.

To assess functional relationships within the three *CsPP2* paralogs, we silenced each gene individually via VIGS in sweet orange. Silencing of *CsPP2-3*, *CsPP2-5*, or *CsPP2-18* significantly reduced target transcript levels but triggered compensatory upregulation of at least one paralog (Fig. 10A), indicating transcriptional redundancy among these genes. Given the inability to culture *C*Las, we used *Xanthomonas citri* subsp. *citri* (*Xcc*), the causal agent of citrus canker, as a proxy to evaluate bacterial resistance. Silencing individual *CsPP2* genes (3-VIGS, 5-VIGS, or 18-VIGS) did not alter *Xcc* proliferation relative to controls (CK) across all timepoints (1–9 days post inoculation, dpi; Fig. 10B), suggesting that basal resistance is maintained through functional compensation by unsilenced paralogs. Conversely, transient overexpression of *CsPP2-3* (3-OE), *CsPP2-5* (5-OE), or *CsPP2-18* (18-OE) in leaves significantly enhanced resistance to *Xcc*. Overexpressing lines exhibited reduced necrotic lesions (Fig. 10C and D) and lower bacterial populations (Fig. 10E) at 9 dpi compared to CK. These complementary results demonstrate that elevating any single paralog is sufficient to confer resistance, while silencing individual genes is buffered by transcriptional compensation, confirming functional redundancy in *Xcc* defense.

### Evaluation of *C*Las resistance in citrus hairy roots overexpressing *CsPP2-3/5/18*

**Figure 11:**
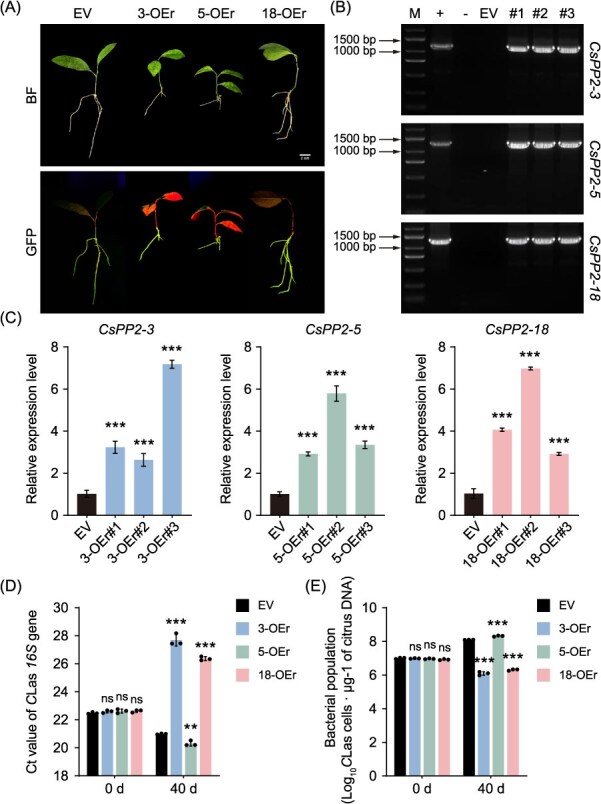
Functional analysis of *CsPP2–3/5/18* on *C*Las resistance in transgenic hairy roots of *C. aurantifolia*. (A) Phenotypes of empty vector control (EV, 1300-EGFP), and *CsPP2–3/5/18*-overexpressing hairy roots under BF and GFP fluorescence. Scale bar, 1 cm. (B) Genomic PCR validation of transgenic hairy roots using CaMV *35S*/gene-specific primers. +, plasmid (positive control); −, ddH_2_O (negative control); EV, empty vector (1300-EGFP); #1–3, independent transgenic lines. (C) Relative transcript levels of *CsPP2–3/5/18* in transgenic roots versus EV roots. (D) Quantification of *C*Las bacterial load based on the cycle threshold (Ct) values of the *C*Las *16S* rDNA gene. (E) *C*Las bacterial titers calculated from the *16S* rDNA gene copy number. Data are presented as mean ± SD (*n* = 3 biological replicates). Asterisks indicate significant differences compared to EV. (Tukey’s test; ^**^, *P* < 0.01; ^***^, *P* < 0.001). ns, not significant.

To investigate the role of *CsPP2-3/5/18* in HLB and to enable rapid assessment of *C*Las resistance, we employed the *A. rhizogenes*-mediated hairy root system. The three candidate genes, *CsPP2-3*, *CsPP2-5*, and *CsPP2-18*, were independently overexpressed in *C*Las-infected lime (*C. aurantifolia*) branches, resulting in the successful regeneration of transgenic hairy roots (Fig. 11A). Genomic PCR and qRT-PCR confirmed successful transformation and effective overexpression in the positive lines (Fig. 11B and C). Subsequent qPCR quantification of *C*Las titer revealed distinct resistance phenotypes. Overexpression of *CsPP2-3* or *CsPP2-18* significantly reduced *C*Las accumulation, as indicated by higher Ct values and lower bacterial populations compared to the control (EV). Conversely, overexpression of *CsPP2-5* led to a marked increase in *C*Las titer, unveiling a susceptibility phenotype (Fig. 11D and E).

## Discussion

PP2 is a central executor of phloem-based defense (PBD), with its massive deposition in sieve tubes during *C*Las infection, considering as a defense mechanism against pathogens in citrus. Despite its pathological significance, the specific PP2 members driving this process and their regulatory logic remained elusive. In this study, through genome-wide identification of 26 *CsPP2* genes in *C. sinensis* and systematic functional dissection, we pinpoint three phloem-specific paralogs (*CsPP2-3/5/18*) as primary mediators of HLB-induced protein deposition. Crucially, these genes exhibit functional redundancy yet mechanistic divergence, revealing an evolved strategy for robust defense without fitness penalty. This work deciphers the molecular architecture of citrus PBD and provides actionable targets for HLB resistance engineering.

### Evolutionary expansion and structural innovation of *CsPP2s*

Our study identified 26 *CsPP2* genes in *C. sinensis*, which were phylogenetically categorized into two distinct subgroups (Group A and Group B) with conserved protein architectures within each group (Fig. 1). All CsPP2s harbor a C-terminal PP2 domain, a hallmark of this protein family, comprising four functionally critical motifs (motifs 2–5; Fig. 1D). These motifs contain highly conserved amino acid residues, such as Glu^2^ and Val^3^ in motif 3, Glu^11^ and Gly^15^ in motif 4, and Lys^8^ and Leu^15^ in motif 5 (Fig. S1). In contrast, the N-terminal region exhibits significant divergence, likely facilitating functional diversification through domain accretion [11]. For instance, the TIR domain, associated with plant immune receptors, is found in the N-terminal region of AtPP2 proteins in *Arabidopsis* [34]. Intriguingly, 21 CsPP2 proteins harbor an N-terminal F-box domain (Fig. 1C and D), suggesting a role in ubiquitin-mediated protein degradation [35]. This prevalence aligns with observations in other species, 12 of 47 GhPP2s in cotton (*Gossypium hirsutum*) and 6 of 15 BnPP2s in ramie (*Boehmeria nivea* L. *Gaudich*) contain N-terminal F-box domains [36, 37]. This domain architecture implies that CsPP2s may regulate protein turnover in pathways, such as stress response or development.

Gene duplication events, involving the replication of genes or gene segments, are the major driver of genome evolution [38]. In this study, we identified three segmental and two tandem duplication events among the 26 *CsPP2* genes (Fig. 2A), indicating gene duplication emerged as a key driver of CsPP2 family expansion. Interestingly, all six duplicated *CsPP2s* (*CsPP2-1*/*2*/*3*/*7*/*17*/*18*) belong to Group A, suggesting that Group A *CsPP2s* represent ancestral lineages whose duplications underpinned the early proliferation of this gene family in *C*. *sinensis*. Genomic collinearity analysis demonstrated stronger synteny between *C. sinensis* and the monocot *O. sativa* in *PP2* genes organization than between *C. sinensis* and the dicot *A. thaliana* (Fig. 2B). Similar synteny patterns were observed in the *DREB* family of Moso bamboo (*Phyllostachys edulis*) [39]. This reinforces the hypothesis that monocots and dicots share common ancestral genes prior to their divergence [40].

### Transcriptional reprogramming of *CsPP2s* in response to citrus HLB

As a phloem-restricted pathogen, *C*Las triggers aberrant phloem protein deposition during infection [31]. However, research on citrus phloem proteins, particularly the specific PP2 family members involved in HLB response and their regulatory mechanisms, remains limited. Our expression profiling revealed seven *CsPP2* genes (*CsPP2-1*, *CsPP2-2*, *CsPP2-3*, *CsPP2-5*, *CsPP2-18*, *CsPP2-22*, and *CsPP2-26*) that were upregulated upon *C*Las challenge, with key candidates exhibiting remarkable induction. For example, *CsPP2-1* and *CsPP2-18* exhibited pronounced 86-fold and 11.13-fold upregulation, respectively (Fig. 5; Table S5). This robust transcriptional activation strongly implicates these *CsPP2* genes in mediating phloem protein deposition during HLB pathogenesis. Promoter *cis*-acting element analysis further revealed that all upregulated *CsPP2* genes contain at least one ABRE and MYC TF-binding element, while all except *CsPP2-26* harbored one or more MYB TF-binding elements (Fig. 3; Table S3). Correspondingly, the predicted upstream regulators of these *CsPP2* genes were predominantly MYB, bHLH (the family of MYC TFs), and bZIP (the family of ABF TFs) (Fig. 4; Table S4). Notably, *CsPP2-18*, which contains the highest number of promoter *cis*-acting elements (18) among upregulated genes, featured predicted regulators including bHLH (9), MYB (8), AP2 (4), and bZIP (4), aligning closely with both the type and abundance of *cis*-acting elements in its promoter. Collectively, these results indicate that HLB-induced *CsPP2* genes likely contribute to the host response, with their expression tightly coordinated by upstream regulatory networks.

### Functional diversification and pathogen-specific modulation of defense by *CsPP2* paralogs

Plant phloem sap, rich in sugars, proteins, and amino acids, is a highly attractive target for pests and pathogenic bacteria [41]. Infection disrupts phloem sieve tubes, causing nutrient loss, impaired translocation, and heightened susceptibility to pathogenic microorganisms [42]. Crucially, the PBD response serves as a core plant defense mechanism against these threats, primarily through phloem protein deposition [43]. Numerous studies have shown that *C*Las infection induces phloem protein accumulation, which blocks sieve pores in citrus leaf veins and restricts the spread of *C*Las. However, specific PP2 members involved in this defense response during HLB infection remain poorly understood. Three *CsPP2* promoters (*CsPP2-3/5/18pro*) with exclusive phloem activity were identified via *A. rhizogenes*-driven GUS assays in citrus hairy roots (Fig. 6C–E). This phloem-restricted expression pattern demonstrates their tissue-specific activity and functional specialization in phloem biology. Notably, all three phloem-localized *CsPP2* genes exhibited significant upregulation upon *C*Las infection (Fig. 5). By contrast, the promoter of *CsPP2-1*, despite being strongly induced by *C*Las, showed no phloem-specific GUS activity even under infection conditions (Fig. S3), suggesting that *CsPP2-1* likely contributes to HLB defense through a non-phloem regulatory pathway.

Our study reveals that the phloem-specialized paralogs *CsPP2-3/5/18* orchestrate a complex, pathogen-specific defense strategy in citrus. While all three genes promote phloem protein deposition and exhibit functional redundancy against the apoplast-colonizing bacterium *Xcc*, they display striking functional divergence against the phloem-restricted *C*Las. This pathogen-specific functional specialization underscores the sophistication of the PP2-based defense network. The most compelling evidence of this specialization comes from the paradoxical behavior of *CsPP2-5*. Overexpression of *CsPP2-5* conferred robust resistance to *Xcc* but unexpectedly enhanced susceptibility to HLB, as evidenced by significantly higher *C*Las titers in transgenic roots (Figs 10 and 11). This contrasts sharply with *CsPP2-3* and *CsPP2-18*, which enhanced resistance against both pathogens. We propose that this divergence reflects a fundamental conflict between physical barrier-based defense and the lifestyle of an endophytic pathogen. Although *Xcc* primarily resides in apoplastic spaces, emerging evidence indicates that phloem-specific genes can coordinate systemic immune responses, thereby influencing pathogen proliferation in distal tissues [44, 45]. Against *Xcc*, the rapid and intensive phloem occlusion driven by CsPP2-5 likely acts as a preemptive physical barrier (Fig. 9F), restricting pathogen spread and potentially facilitating the defense signal propagation, thereby conferring *Xcc* resistance despite the downregulation of some defense-related genes. In the case of *C*Las, however, the same mechanism may prove detrimental. We hypothesize that the dense protein plugs formed by *CsPP2-5* overexpression trap nutrients within the sieve elements, creating a protected, nutrient-rich niche that inadvertently promotes *C*Las multiplication and shields it from host immune mechanisms. In this scenario, the simultaneous downregulation of SA-mediated defense genes by *CsPP2-5* may further cripple the chemical immunity needed to eliminate the entrapped pathogen (Fig. 9G).

This functional redefinition of *CsPP2-5* illuminates the previously enigmatic role of its counterpart, *CsPP2-B15*, which was recognized as one of the most highly upregulated genes during HLB infection but whose function remained opaque [23]. Our data now position *CsPP2-5* (*CsPP2-B15*) not merely as a marker of pathology, but as a central regulator in a defense trade-off. Its pronounced induction during natural HLB infection may represent a host attempt to wall off the pathogen, a strategy that, in susceptible varieties, is ultimately exploited by *C*Las for its own benefit. Conversely, *CsPP2-3* and *CsPP2-18* promote a chemical immunity strategy. Their overexpression consistently activated systemic acquired resistance (SAR) markers and SA-responsive genes, establishing a broad-spectrum resistance that is effective against both *Xcc* and *C*Las. The functional divergence within the *CsPP2* family reflects an evolutionary-based ‘division of labor’: *CsPP2-5* primarily facilitates physical containment, whereas the *CsPP2-3* and *CsPP2-18* sustain chemical defense signaling. Such specialization enables the host to deploy layered defenses, balancing the costs and benefits of different strategies to combat diverse pathogens. The retention of these three paralogs with overlapping yet distinct functions equips citrus with a resilient and adaptable immune toolkit, crucial for navigating the challenges posed by co-evolving pathogens such as *Xcc* and *C*Las.

In conclusion, our genome-wide identification of *PP2* genes in *C. sinensis* uncovered three phloem-specialized paralogs (*CsPP2-3/5/18*) that drive phloem protein deposition. We demonstrated their functional redundancy in disease resistance alongside molecular specialization in immune signaling. Their structural divergence and unique roles in immune signaling may enable them to achieve flexible multi-layered defense, an adaptive strategy that allows citrus to balance growth and defense costs while combating evolving pathogens.

## Materials and methods

### Plant cultivation and phytohormone treatments

Two-month-old sweet orange (*C*. *sinensis*) seedlings were cultivated in a greenhouse at the National Navel Orange Engineering Research Center (Gannan Normal University). Plants were maintained under controlled conditions (25 °C with a 16-h light/8-h dark photoperiod). For tissue-specific expression analysis, whole seedlings were carefully uprooted, rinsed to remove soil, and separated into leaves, stems, and roots. For phytohormone treatments, plants received either root drenching or foliar spraying with 50 mL of the following solutions per plant: 100 mM ABA, 20 mg/L ETH, 5 mg/L GA, 200 μM JA, or 500 μM SA, respectively [46–50]. Leaf samples were collected at 0, 3, 6, 12, and 24 h post-treatment, with three biological replicates for each time point. All samples were immediately frozen in liquid nitrogen and stored at −80 °C for subsequent RNA extraction and gene expression analysis.

### Identification and characterization of the *PP2* genes in *C*. *sinensis*

The HMM of the conserved PP2 domain (PF14299) was retrieved from the Pfam database (http://pfam.xfam.org/) and used as a query to conduct an HMMER search (http://hmmer.janelia.org/, Version 3.0) against the *C. sinensis* proteome (http://citrus.hzau.edu.cn/index.php) with an *E*-value cutoff of 1*e*-5 to identify potential PP2 proteins [51]. Candidate sequences were validated for complete PP2 domains using Conserved Domain Database (http://www.ncbi.nlm.nih.gov/Structure/cdd/wrpsb.cgi) and SMART (http://smart.embl-heidelberg.de/) [52, 53]. Redundant isoforms and partial-domain sequences were excluded, with non-redundant proteins designated as definitive CsPP2 genes. Molecular weight (MW) and pI of the identified CsPP2 proteins were predicted using ExPASy (https://web.expasy.org/compute_pi/) [54].

### Phylogenetic analysis, conserved domain identification, and structural organization

PP2 protein sequences of *A*. *thaliana* were obtained from TAIR (The Arabidopsis Information Resource; http://www.arabidopsis.org/) [55]. Multiple sequence alignment of *C. sinensis* and *Arabidopsis* PP2 homologs was performed using ClustalW (https://www.genome.jp/tools-bin/clustalw, Version 2.0) [56]. A neighbor-joining phylogenetic tree was constructed in MEGA software (https://www.megasoftware.net/, version 11.0) with 1000 bootstrap replicates to evaluate node support [57]. Gene structures (intron/exon organization) of *CsPP2s* were visualized using the ‘Gene Structure View’ module in TBtools software (version 2.083) based on the genomic GFF file of *C. sinensis* [58]. Conserved motifs were identified via MEME (http://meme-suite.org/tools/meme, Version 5.5.5) with the following parameters: a maximum of motifs set to 6, and an optimal length ranging from 6 to 100 amino acids [59].

### Chromosomal distribution, gene duplication, and synteny analysis

Duplication events of the *CsPP2s* were analyzed using the ‘MCScanX’ module of TBtools software, and the results were visualized using the ‘Advanced Circos’ module. Based on the chromosomal localization information in the *C. sinensis* genomic GFF annotation, *CsPP2* genes were mapped to the nine chromosomes. Genes were arranged in ascending order of their physical positions, from short-arm to long-arm telomeres [58]. Genomic sequence and annotation files for Arabidopsis and rice (*O. sativa* subsp. *japonica*) were downloaded from the Ensembl database (https://www.ensembl.org) [60]. Syntenic blocks were identified and visualized using the ‘Dual Synteny Plot’ module in TBtools.

### *cis*-Acting element and TF regulatory network analysis of *CsPP2* genes

The 2.5-kb upstream sequences of the TSS of 26 *CsPP2* genes were extracted using the ‘GTF/GFF3 Sequence Extract’ module in TBtools. *Cis*-acting elements were identified through PlantCARE (http://bioinformatics.psb.ugent.be/webtools/plantcare/html/) [61] and visualized using the ‘Simple Biosequence Viewer’ module in TBtools. Potential TFs binding to *CsPP2* promoter were predicted via PlantRegMap (https://plantregmap.gao-lab.org/binding_site_prediction.php), with a significance threshold of *P*-value <1*e*-5. *C*. *sinensis* was selected as the target species for analysis [62]. The predicted TFs regulatory networks were constructed in Cytoscape (v3.9.1) [63]. TF family enrichment was visualized as wordcloud using the ggplot2 package in R [64], and the heatmaps were established using TBtools based on log_2_-transformed expression values.

### DNA extraction and *C*Las quantification

Leaf samples from asymptomatic and HLB-symptomatic sweet orange or lime plants were collected from orchard. Midrib or petiole tissue (100 mg) were lyophilized, pulverized, and subjected to genomic DNA (gDNA) extraction using the cetyltrimethylammonium bromide (CTAB) method. The gDNA concentration was quantified using a NanoDrop 2000 spectrophotometer (NanoDrop Technologies, Wilmington, DE, USA) and diluted to a working concentration of 20 ng/μL using sterile distilled water. To quantify *C*Las infection levels, the *16S* ribosomal RNA gene of *C*Las was amplified using q*C*Las *16S* F/q*C*Las *16S* R primers according to the protocol described by a previous study [65]. Each 20 μL qPCR reaction contained 20 ng of gRNA as template. Based on the Ct value of the *C*Las *16S* gene, samples with Ct < 31.3 were determined as *C*Las positive, while those with Ct > 36 were considered *C*Las negative. All samples were analyzed with three biological and three technical replicates to ensure data reliability.

### RNA extraction and qRT-PCR analysis

Total RNA was isolated using the OminiPlant RNA Kit (CWBIO, China) following the manufacturer’s instructions. RNA integrity was confirmed by agarose gel electrophoresis and concentration was measured using a NanoDrop 2000 spectrophotometer. High-quality RNA was reverse-transcribed into cDNA using the PrimeScript™ RT kit with gDNA Eraser (Takara, Japan). Primers specific for *CsPP2s* and defense-related genes were designed using the ‘Batch qPCR Primer Design’ module in TBtools and listed in Table S6. qRT-PCR was conducted on a QuantStudio 5 Applied Biosystem (ThermoFisher Scientific, USA) with 2 × TSINGKE^®^ Master qPCR Mix (TSINGKE, China). The *Actin* gene of *C*. *sinensis* was employed as an internal reference for normalization. Relative expression levels were calculated using the 2^-ΔΔCt^ method, with three biological replicates and three technical replicates for each sample.

### Hairy roots transformation

For promoter expression analysis, ~2.5 kb promoter of 26 *CsPP2s*, *35S,* and *CsSUC2pro* (phloem-specific positive control), along with truncated versions of *CsPP2-3/5/18* (designated F1-F3), were cloned into the DX2181G-EGFP vector at *Hind* III/*Bam*H I sites. For overexpression, full-length *CsPP2-3/5/18* CDSs were inserted into pCAMBIA1300-EGFP at *Xba* I/*Xma* I sites. Constructs and empty vectors were individually transformed into *A. rhizogenes* K599. Positive *Agrobacterium* suspensions were incubated to an OD_600_ of 0.6 and resuspended in 2-(N-morpholino) ethanesulfonic acid (MES) buffer (10 mM MgCl_2_, 10 mM MES, and 100 μM acetosyringone, pH 5.6) to prepare the infiltration solution. Germinated sweet orange seeds were immersed in the infiltration solution and subjected to vacuum infiltration for 10 min at 0.08 to 0.09 MPa. Subsequently, the transformed seeds were incubated in the dark for 3 days and then transferred to vermiculite trays. Hairy roots were abundantly observed 30 days post-inoculation. Transgenic roots were preliminarily screened for fluorescence using a portable excitation light source (Luyor-3415RG, Shanghai, China). Detailed protocols for *A. rhizogenes*-mediated hairy root transformation followed the method described by a previous research [32].

### GUS staining and microscopic examination

Hairy roots (wild-type and transgenic lines harboring *35S*, *CsSUC2pro*, 26 *CsPP2s* promoters, or truncated *CsPP2-3/5/18* promoter fragments) were subjected to GUS staining using a histochemical assay kit (RTU4032, Real-Times Biotechnology Co. Ltd, Beijing, China) according to the manufacturer's protocol. Stained roots were paraffin-embedded and sectioned. GUS signal distribution in cross-sections was visualized using a fluorescence microscope (Leica DM3000, Germany).

### Subcellular localization analysis

The coding sequences (without stop codons) of *CsPP2-3*, *CsPP2-5*, and *CsPP2-18* were cloned into the pBI121-EGFP vector at *Xba* I and *Xma* I sites. Fusion plasmid and EV were introduced into *A. tumefaciens* GV3101, and subsequently used for transient expression in *Nicotiana benthamiana* leaves. After 3 days of incubation, green fluorescence signals were observed using a confocal laser scanning microscope (Leica TCS SP8, Germany). *Agrobacterium*-mediated transformation and leaf infiltration were performed according to the method described by a previous study [66].

### Microstructure and TEM observation of the phloem

Phloem thickness in overexpressing hairy roots was quantified from ≥10 independent lines per group using ImageJ software. For TEM observation, the transgenic roots were cut into 1 mm lengths and fixed in 2.5% glutaraldehyde (pH 7.0) for 2 h at room temperature, followed by overnight fixation at 4°C. Samples were rinsed three times with 0.1 M phosphate buffer (PB; pH 7.4) for 15 min each, followed by dehydration in alcohol at room temperature and osmotic embedding in acetone. Embedded blocks were sectioned into 60- to 80-nm slices using an ultrathin sectioning machine (Leica UC7, Germany). Phloem protein deposition was observed using a transmission electron microscope (HITACHI HT7800, Japan).

### VIGS vector construction, transient transformation, and *Xcc* inoculation

Gene-specific fragments (targeting non-conserved regions of *CsPP2-3/5/18*) were PCR-amplified and individually cloned into the pTRV2 vector. Recombinant plasmids (pTRV2-*CsPP2-3/5/18*) and the empty pTRV2 control were transformed into *A. tumefaciens* GV3101. Bacterial cultures harboring each construct and pTRV1 (helper plasmid) were grown to OD_600_ = 0.8, harvested, and resuspended in infiltration buffer (10 mM MES, 10 mM MgCl₂, 200 μM acetosyringone). For VIGS, suspensions of pTRV1 and each pTRV2-*CsPP2* recombinant (or pTRV1 + empty pTRV2 control) were mixed 1:1 (v/v), incubated in darkness for 2 to 3 h, and co-infiltrated into the abaxial side of sweet orange leaves. The left and right halves of each leaf received the control (pTRV1 + empty pTRV2) and experimental (pTRV1 + pTRV2-CsPP2) solutions, respectively. For transient overexpression, *CsPP2-3/5/18*-pCAMBIA1300-EGFP constructs and the EV control were similarly transformed into GV3101, and bacterial suspensions were prepared identically. For each group, six to eight leaves were injected and cultured in the dark for 3 days. and then samples were collected for qRT-PCR analysis. Subsequently, all infiltrated leaves were inoculated with *Xcc* by injecting a bacterial suspension (10^6^ CFU/mL) into both the control and experimental zones of each leaf half. Inoculated plants were transferred to a growth chamber (28 °C, 16-h light/8-h dark cycle). Bacterial titers within infiltrated zones were quantified at 1, 3, 5, 7, and 9 dpi.

### Evaluation of *C*Las resistance based on the hairy root transformation system

*C*Las-positive lime branches were collected and used for hairy root transformation via the *A. rhizogenes*-mediated method, as previously established. The *CsPP2-3/5/18*-pCAMBIA1300-EGFP overexpression vectors were individually introduced into the infected branches. An empty vector was transformed into *C. aurantifolia* branches with similar *C*Las titers to serve as the control. At 40 days post-transformation, the regenerated transgenic roots were assessed by fluorescence observation, genomic PCR identification, and relative expression analysis. The bacterial titer of *C*Las in the transgenic hairy roots was quantified via qPCR targeting the *16S* rDNA gene. All experiments included three biological replicates, each with three technical replicates.

### Statistical analysis

Data were evaluated using Tukey's multiple comparison test in the ANOVA program of the SAS software package (SAS Institute, Cary, NC, USA). Statistical significance was considered at *P* < 0.05.

## Supplementary Material

Web_Material_uhaf333

## Data Availability

All data supporting the findings in this study are available in the manuscript or Supplementary Data.
