# Supplementary material for: Genome-wide dissection of PP2 genes reveals CsPP2-3/5/18 as key regulators of phloem protein deposition and bacterial immunity in Citrus sinensis
Source: Hortic Res. 2025 Dec 4;13(3):uhaf333. doi: 10.1093/hr/uhaf333 (PMC12966013; doi:10.1093/hr/uhaf333)
Supplement: Web_Material_uhaf333 [file web_material_uhaf333.zip › Supplementary figures.pdf]

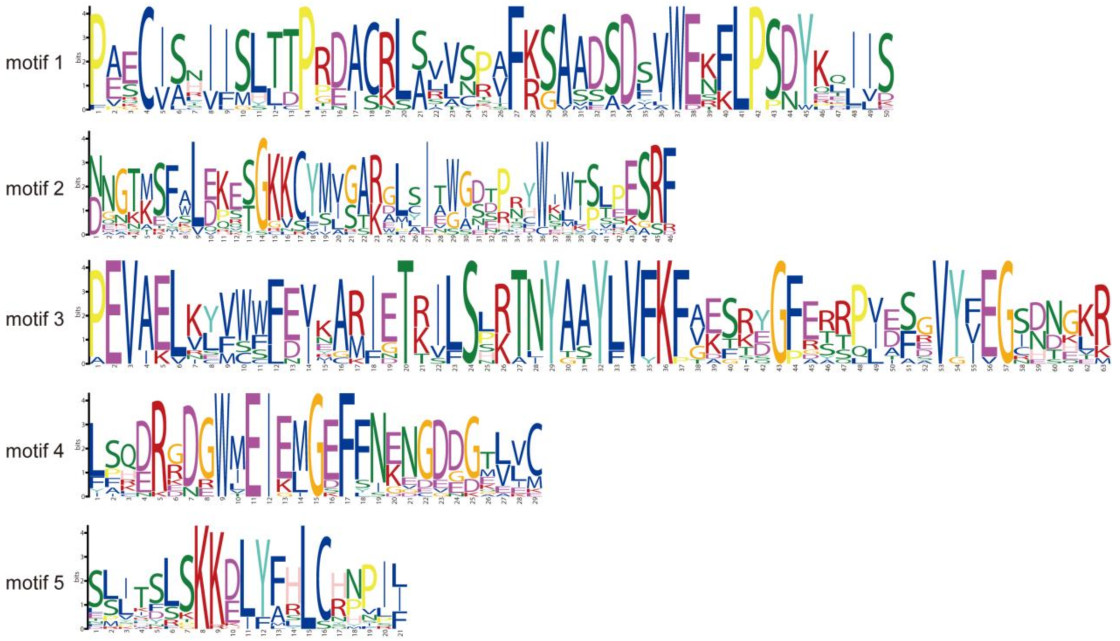

Figure S1. Conserved domain architecture of CsPP2 proteins.

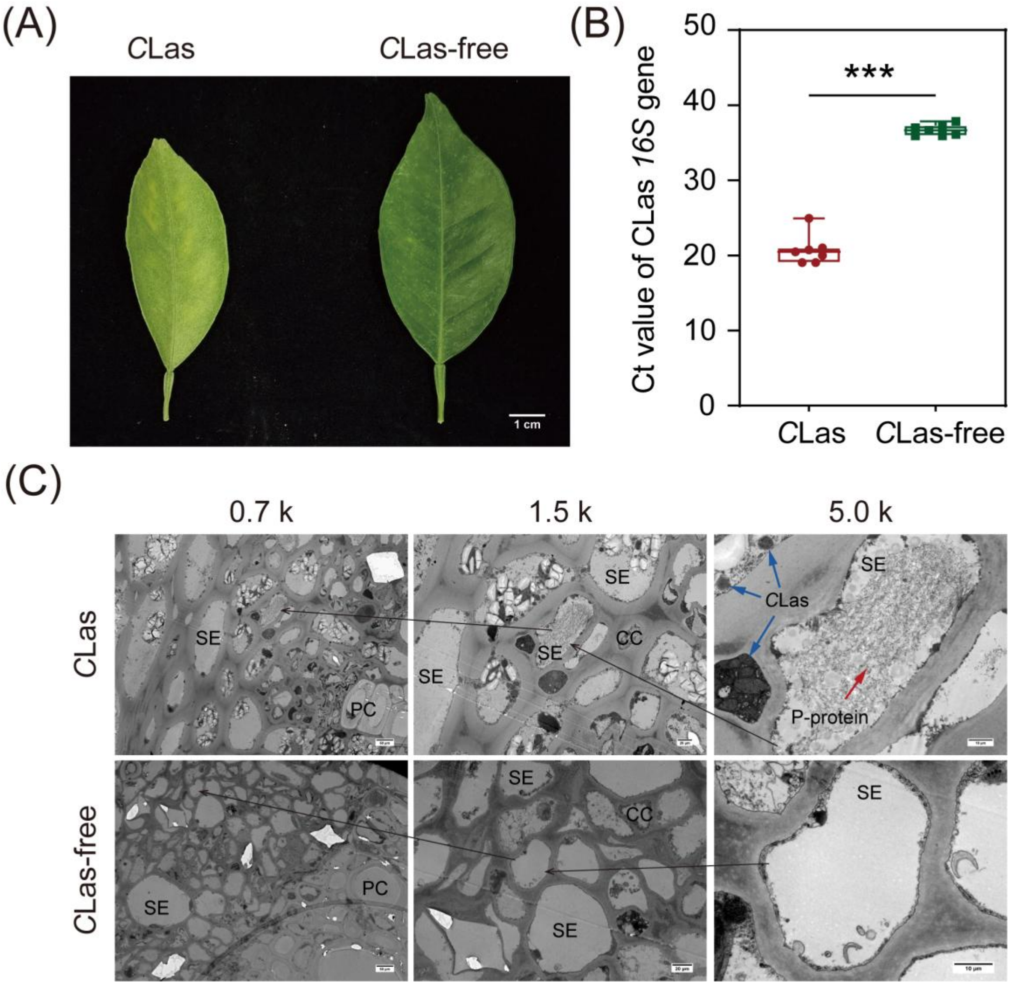

Figure S2. Phloem protein deposition in healthy and *C*Las-infected *C. sinensis* leaves. (A) Phenotypes of healthy (*C*Las-free) and *C*Las-infected (*C*Las) sweet orange leaves. Scale bar = 1 cm. (B) Cycle threshold (Ct) values of the *C*Las *16S* rDNA gene in midribs of healthy and infected leaves. The extracted gDNA was quantified with a NanoDrop spectrophotometer, and 20 ng of gDNA was used as the template in each qPCR reaction to quantify the level of *C*Las 16S rDNA. Asterisks indicate significant differences (Tukey's test; ***, $p < 0.001$). (C) TEM images of phloem protein deposition in healthy and *C*Las-infected leaf veins. Red arrow: phloem protein (P-protein) deposits; blue arrows: *C*Las. PC: parenchyma cells; SE: sieve element; CC: companion cells.

(A)

NO.	Ct value of *C*Las *16S* gene
1	29.125
2	28.302
3	21.807
4	26.331
5	21.869
6	29.862
7	25.254
8	21.600
9	23.305
10	25.201
Positive	21.212
Healthy	36.997
ddH_2O	Undetermined

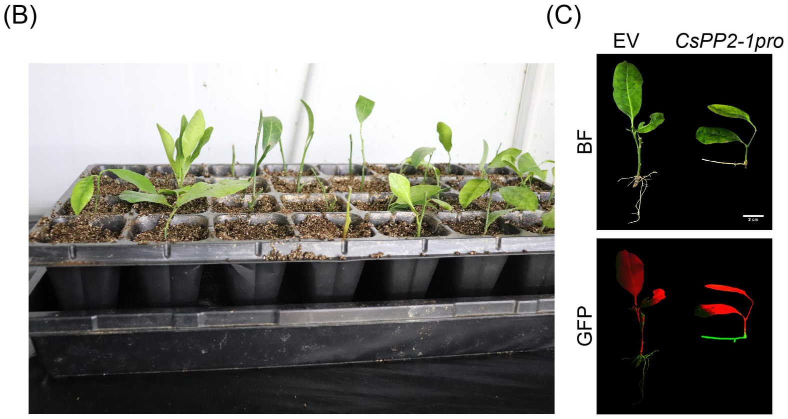

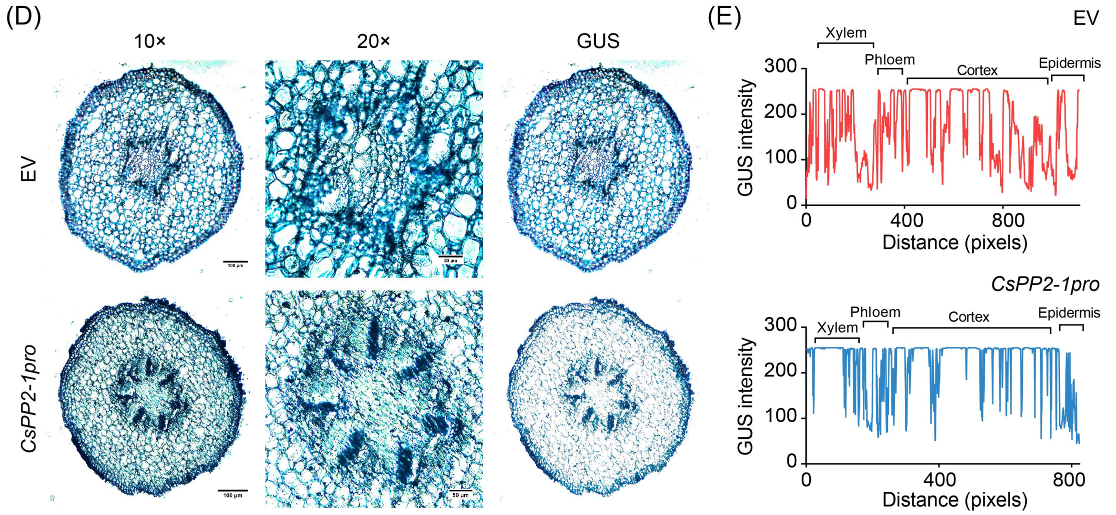

Figure S3. Evaluation of *CsPP2-1* promoter activity in *C*Las-infected *C. aurantifolia* branches. (A) Ct values of the *C*Las *16S* rDNA in individual branches. Samples highlighted in red, which exhibited low and uniform Ct values, were selected for subsequent hairy root induction. (B) Hairy root cultures derived from *C*Las-infected stems following *A. rhizogenes* transformation. (C) Representative images of hairy roots emerging from a *C*Las-infected lime branch at 40 d post-induction. Bright field (BF) and GFP fluorescence views are shown for *35S*::GUS and *CsPP2-1pro*::GUS transgenic hairy roots. Scale bar = 1 cm. (D) High-magnification views of GUS staining (10×: 100 μm scale; 20×: 50 μm scale). Right panels: ImageJ-isolated GUS signals (blue) from 10× views. (E) Quantification of GUS activity in vascular tissues.
